# Do the socioeconomic impacts of antiretroviral therapy vary by gender? A longitudinal study of Kenyan agricultural worker employment outcomes

**DOI:** 10.1186/1471-2458-9-240

**Published:** 2009-07-15

**Authors:** Bruce A Larson, Mathew P Fox, Sydney Rosen, Margret Bii, Carolyne Sigei, Douglas Shaffer, Fredrick Sawe, Kelly McCoy, Monique Wasunna, Jonathan L Simon

**Affiliations:** 1 Department of International Health, Boston University School of Public Health, 801 Massachusetts Ave, Crosstown 3rd Floor, Boston, MA 02118, USA; 2 Center for International Health and Development, Boston University School of Public Health, 801 Massachusetts Ave, Crosstown 3rd Floor, Boston, MA 02118, USA; 3 Kenya Medical Research Institute, Hospital Road, BO Box 1357-20200, Kericho, Kenya; 4 United States Army Medical Research Unit-Kenya, Walter Reed Project, Hospital Road, BO Box 1357-20200, Kericho, Kenya; 5 Kenya Medical Research Unit, P.O. Box 20778, 00200, Nairobi, Kenya

## Abstract

**Background:**

As access to antiretroviral therapy (ART) has grown in Africa, attention has turned to evaluating the socio-economic impacts of ART. One key issue is the extent to which improvements in health resulting from ART allows individuals to return to work and earn income. Improvements in health from ART may also be associated with reduced impaired presenteeism, which is the loss of productivity when an ill or disabled individual attends work but accomplishes less at his or her usual tasks or shifts to other, possibly less valuable, tasks.

**Methods:**

Longitudinal data for this analysis come from company payroll records for 97 HIV-infected tea estate workers (the index group, 56 women, 41 men) and a comparison group of all workers assigned to the same work teams (n = 2485, 1691 men, 794 women) for a 37-month period covering two years before and one year after initiating ART. We used nearest neighbour matching methods to estimate the impacts of HIV/AIDS and ART on three monthly employment outcomes for tea estate workers in Kenya – days plucking tea, days assigned to non-plucking assignments, and kilograms harvested when plucking.

**Results:**

The female index group worked 30% fewer days plucking tea monthly than the matched female comparison group during the final 9 months pre-ART. They also worked 87% more days on non-plucking assignments. While the monthly gap between the two groups narrowed after beginning ART, the female index group worked 30% fewer days plucking tea and about 100% more days on non-plucking tasks than the comparison group after one year on ART. The male index group was able to maintain a similar pattern of work as their comparison group except during the initial five months on therapy.

**Conclusion:**

Significant impaired presenteeism continued to exist among the female index group after one year on ART. Future research needs to explore further the socio-economic implications of HIV-infected female workers on ART being less productive than the general female workforce over sustained periods of time.

## Background

The negative impacts of untreated HIV/AIDS on individuals and households in sub-Saharan Africa have been documented in a range of settings and countries [1-6]. In the absence of antiretroviral therapy, studies from resource-limited settings suggest a median survival time of 11 months following an AIDS diagnosis [7]. With nearly 3 million people receiving antiretroviral therapy (ART) as of December 2007 [8], attention has turned to evaluating the socio-economic impacts of treatment of HIV/AIDS on individuals, households, and private sector companies.

The limited evidence available so far on the socio-economic impacts of ART in sub-Saharan Africa is encouraging. Employee work records at large industrial enterprises in Africa indicate that absenteeism of HIV-infected workers declines quickly after they initiated therapy [9-11]. Other studies using self-reported data also find positive impacts of ART in terms of labor force participation, schooling of children whose parents are on ART, and quality of life outcomes for ART patients [12-17]. Although methods and outcome measures differ, each of these studies finds some positive socio-economic impacts of ART during the initial three to twelve months on ART.

An important question for HIV-positive adults and their households is the extent to which improvements in health resulting from ART allow a return to income-generating activities, whether formal sector employment or informal, household-based production such as family farming or informal day labor. Returning to income generating activities includes reducing absenteeism from work and impaired presenteeism, which is the loss of productivity when an ill or disabled individual attends work but either accomplishes less at his or her usual tasks or shifts to less valuable tasks [18-22].

Given the substantially different responsibilities of men and women in sub-Saharan Africa, especially in rural communities, the impact of ART on income-generating activities could vary substantially by gender. Using exceptionally detailed daily payroll data, we conducted a gender-stratified analysis of impaired presenteeism among HIV-infected agricultural plantation workers on ART in Kenya with matched comparison groups from the general workforce over a 37-month period covering two years pre-ART, the month of initiation, and 12 months post-ART.

In this agricultural workforce setting, harvesting less tea when plucking and shifting from harvesting tea to less strenuous, non-plucking tasks are two quantitative measures of impaired presenteeism. We focused on impaired presenteeism because tea pluckers, and plantation employees generally, are likely to face strong pressures to attend work even if feeling unwell due to the need to earn income for their family and fear of job loss. As gender influences work assignments on the plantation and may play a central role in the perception of and responses to such pressures, we stratified our data to explore differences in patterns of absenteeism and impaired presenteeism between males and females.

## Methods

### Study Site

The study site is a large tea plantation located in Kericho District of the southern Rift Valley Province of Kenya. Previous research at a different plantation in this district documented the decline in work performance associated with untreated HIV/AIDS in a treatment-naïve population of tea pluckers [3].

The plantation included in this study has a workforce of more than 10,000 employees on permanent contract and numerous others on temporary arrangements, with the majority of all workers employed as tea pluckers. Tea is harvested in every month of the year. Almost all employees live on the plantation in company-provided family housing.

In 2004, population HIV prevalence in the study area was estimated at 14.1 percent (11.1 percent among men and 18.1 percent among women) [23]. The tea company maintains a central hospital and a group of dispensaries and clinics for providing medical care to workers and their dependents free of charge as part of their employment benefits. Transportation to the hospital is also provided by the company. An HIV/AIDS treatment program was introduced in the region in 2004 through donor funding, and the company hospital began providing ART to employees and dependents free of charge in April 2004 as part of the donor-funded program.

Our study was restricted to permanent employees defined as tea pluckers in the company's human resource records. On days spent plucking tea, workers are paid a fixed rate per kilogram (kg) harvested. In 2005, the pay rate per kilogram was 5.44 Kenyan Shillings (KES) ($1 = KES 75.71 in 2005). Baskets of tea are weighed electronically in weighing stations located near fields to measure quantities harvested by each worker.

On some days tea pluckers are instead assigned to non-plucking tasks (*e.g. *weeding, pruning, maintenance activities). These tasks are compensated at a flat daily rate equivalent to plucking 34 kgs of tea (KES 185 in 2005, $2.44). This pay structure provides positive incentives for workers to pluck tea because healthy workers typically harvest more than 34 kg of tea per day. Sick workers who cannot pluck 34 kgs, however, can engage in non-plucking assignments to remain at work for that day, earn income, and mask at least to some degree their poorer performance (a form of impaired presenteeism). A field supervisor can assign pluckers to non-plucking tasks based on operational need, the perceived physical condition of the worker, the worker's request, or the company hospital's instructions.

Ethics approval was obtained from Boston University (H-25060), the Kenya Medical Research Institute (SSC #993), and the Walter Reed Army Institute for Research (WRAIR #1247).

### Selection of Index and Comparison Groups

The index group consisted of 97 permanently employed HIV-infected adult tea pluckers (41 men, 56 women) who initiated ART between April 2004 and May 2006 and were hired at least 24 months before initiating ART. Payroll data are available through May 2007, so a minimum of 37 months of employment data (approximately 800 daily observations) exists for each of these workers (24 months before initiating ART, the month of ART initiation, and 12 full months on ART).

Tea pluckers work in groups known as gangs, with pluckers in the same gang working in the same fields on the same estates on the same days. The comparison groups for this analysis were selected from the general workforce comprised of all permanently employed tea pluckers who were assigned to the same gangs as workers in the index group and were also hired at least 24 months prior to the date when the index worker in that gang initiated ART. This general workforce included 2,485 workers (1,691 men and 794 women). The exact male and female comparison group drawn from this general workforce is explained below under methods.

### Data Development and Definition of Outcomes

We collapsed the daily payroll data (daily work activities and quantities of tea plucked) into monthly units of observation with the following outcomes for each study subject: (1) the number of days spent plucking tea per month; (2) the number of days on non-plucking assignments per month; and (3) average daily output when plucking per month. A monthly unit of analysis is consistent with the pay structure on the plantation and the level of detail evaluated by company management. In this setting, absenteeism is based on total working days (plucking and non-plucking combined), while impaired presenteeism includes both harvesting less when plucking and shifting from plucking to non-plucking assignments.

Based on the date when each individual in the index group began ART, we calculated a "duration-on-ART" time variable for each gang as the difference in months between the calendar date month and the month when the index subject in that gang initiated ART. For example, if the index worker in gang 10 began ART in December of 2005, the duration-on-ART variable for all workers in gang 10 equals 0 for December 2005, -2 for October 2005, and 2 for February 2006. Because workers initiated ART on various days during the month, month 0 is interpreted as being on ART for less than one month.

### Data Analysis

During the pre-ART period (months -24 to -1), the impact of HIV/AIDS disease on work performance would be reflected by plucking on fewer days, harvesting fewer kilograms when plucking, and/or working a different number of days on non-plucking tasks than an appropriate comparison group. Evidence that any monthly differences between the index and comparison groups grew smaller and eventually disappeared during the post-ART period (months 1 to 12) would provide strong evidence on the employment impacts of ART.

To estimate mean differences between the index and comparison groups for each month of the study, we first stratified the analysis by gender to account for differences in the work patterns for men and women. In the general workforce, women typically work more days plucking tea per month than men, men spend more days at non-plucking tasks, and women harvest fewer kilograms per day on days spent plucking.

We then used nearest-neighbour matching methods [24,25] to estimate the difference in mean outcomes for the index group and a matched comparison group for each study month. With nearest neighbor matching, each index subject was matched to four comparison workers from the general workforce for each study month on three observable characteristics – gang, age, and number of months employed. Matching on gang controlled for productivity differences between fields and possible effects of gang supervisors for each study month. Matching on age and duration of employment controlled for the effect of these characteristics on individual productivity (e.g. strength and skill). It was not possible to match exactly on these two continuous variables. For example, the age and months of experience of an index subject might be 41 and 27 (years old, months of experience), but the four closest matches in the same gang and gender could be 41 and 29, 39 and 31, 40 and 27, and 42 and 26. To adjust for this imperfect matching on continuous variables, the bias-corrected version of the matching estimator was used (from STATA 10.0).

## Results and discussion

Table 1 describes the demographic and clinical characteristics of the index groups and general workforce. For both men and women, age and years of experience were similar for the index group and general workforce, with no significant differences. The median CD4 count closest to the date of ART initiation, which was obtained directly from the company hospital for each index subject, was 183 [IQR: 105–207] for the female index group and 144 [IQR: 68–224] for male index group. While men are a larger proportion of the tea plucker work force, HIV prevalence is substantially higher for women in the study region, which is why women are a higher proportion of the index group than the general workforce.

**Table 1:** Descriptive characteristics for index groups and the general workforce

	Men		Women	
		
	General Workforce^/1^	Index Group	General Workforce	Index Group
Number	1691	41	794	56
Age in years^/2^	38.5	38.4	40.6	40.5
Years of Work Experience^/2^	9.8	9.0	10.5	9.9
CD4 count for initiating ART				
Mean		144		183
Median		145		187
IQR		68–224		105–270

/1 The general workforce is defined as all permanently employed tea pluckers assigned to the same work gangs as workers in the index group who were hired at least 24 months before the index worker in their gang initiated ART.

/2 Age and work experience were evaluated as of the month and year that the index worker in each gang initiated ART.

**Figure 1:**
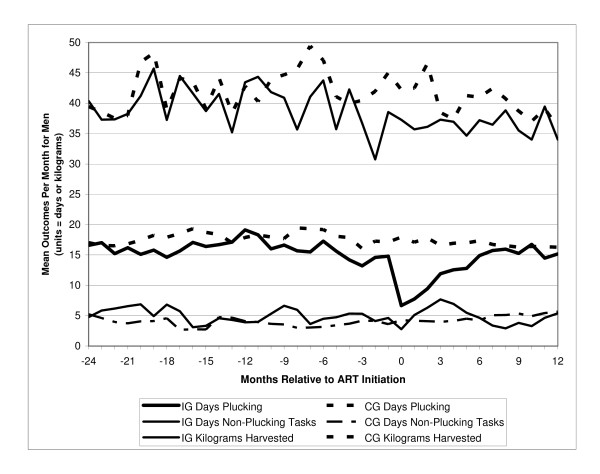
**Mean outcomes by group (men)**. *Data from tables 2 and 4.*

**Figure 2:**
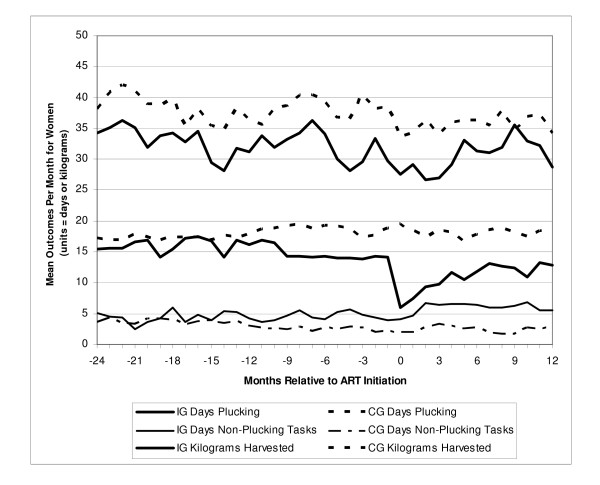
**Mean outcomes by group (women)**. *Data from tables 3 and 4.*

Tables 2, 3, and 4 report monthly means for the three study outcomes: the number of days workers in each index group completed plucking and non-plucking assignments and for kilograms harvested when plucking. Tables 2, 3, and 4 also provide the matching estimator results – estimated mean difference, p-value, and 95% confidence intervals – for each study month for the three outcomes. Figure 1 for men and Figure 2 for women summarize these results for each outcome for each month of the study. In Figures 1 and 2, the index group means are from Tables 2, 3, and 4, and the means for the matched comparison groups are calculated using the index group mean and the estimated mean difference also reported in Tables 2, 3, and 4. We summarize below key results by gender.

**Table 2:** Index group means and matching estimator results for days plucking tea and non-plucking days for men

**Duration on ART**	**N**	**Days Plucking (IG Mean)**^\1^	**Estimated Mean Difference** **(IG – CG)**^\1^	**P > |z|**^\2^	**95% CI LL**^\2^	**95% CI UL**^\2^	**Days Non-Plucking Tasks (IG Mean)**	**Estimated Mean Difference** **(IG – CG)**^\1^	**P > |z|**^\2^	**95% CI LL**^\2^	**95% CI UL**^/2^
-24	41	16.59	-0.43	0.78	-3.42	2.57	4.76	-0.47	0.75	-3.30	2.36
-23	41	17.02	0.46	0.76	-2.49	3.40	5.85	1.26	0.39	-1.58	4.09
-22	41	15.22	-1.30	0.43	-4.50	1.90	6.17	2.22	0.13	-0.64	5.08
-21	41	16.20	-0.62	0.72	-4.03	2.80	6.56	2.82	0.08	-0.31	5.94
-20	41	15.10	-2.33	0.16	-5.58	0.92	6.88	2.81	0.07	-0.24	5.85
-19	41	15.80	-2.41	0.12	-5.48	0.65	4.93	0.82	0.56	-1.90	3.53
-18	41	14.61	-3.30	0.04	-6.37	-0.22	6.83	2.25	0.13	-0.67	5.16
-17	41	15.66	-2.86	0.06	-5.88	0.16	5.73	3.07	0.02	0.54	5.60
-16	41	17.07	-2.24	0.14	-5.24	0.75	3.10	0.32	0.77	-1.81	2.45
-15	41	16.39	-2.34	0.18	-5.74	1.07	3.34	0.61	0.63	-1.85	3.07
-14	41	16.71	-1.66	0.26	-4.57	1.25	4.59	-0.17	0.89	-2.62	2.27
-13	41	17.12	0.13	0.93	-3.05	3.32	4.29	-0.38	0.77	-2.98	2.22
-12	41	19.12	1.24	0.42	-1.79	4.28	3.90	-0.19	0.89	-2.88	2.50
-11	41	18.32	0.03	0.98	-2.82	2.88	3.98	0.02	0.99	-2.66	2.71
-10	41	16.00	-1.97	0.22	-5.12	1.18	5.32	1.67	0.27	-1.28	4.62
-9	41	16.63	-1.06	0.49	-4.08	1.95	6.63	3.07	0.03	0.32	5.81
-8	41	15.68	-3.76	0.02	-6.99	-0.53	5.98	2.96	0.03	0.36	5.56
-7	41	15.49	-3.84	0.02	-7.02	-0.66	3.63	0.59	0.63	-1.81	2.99
-6	41	17.27	-1.90	0.21	-4.90	1.09	4.49	1.31	0.32	-1.28	3.91
-5	41	15.63	-2.46	0.13	-5.63	0.72	4.73	1.32	0.33	-1.32	3.96
-4	41	14.20	-3.66	0.03	-7.02	-0.30	5.34	1.67	0.27	-1.27	4.62
-3	41	13.20	-2.85	0.08	-6.05	0.34	5.32	1.15	0.46	-1.87	4.17
-2	41	14.59	-2.70	0.12	-6.09	0.69	4.12	-0.04	0.98	-2.86	2.77
-1	41	14.80	-2.33	0.14	-5.38	0.73	4.63	1.03	0.48	-1.84	3.91
0	41	6.66	-11.27	0.00	-13.85	-8.69	2.78	-1.48	0.20	-3.74	0.78
1	41	7.76	-9.34	0.00	-12.21	-6.48	5.12	0.95	0.51	-1.88	3.78
2	41	9.44	-8.37	0.00	-11.32	-5.42	6.32	2.22	0.11	-0.52	4.96
3	41	11.90	-4.65	0.00	-7.74	-1.56	7.68	3.68	0.01	0.86	6.51
4	41	12.56	-4.37	0.01	-7.55	-1.19	6.93	2.80	0.04	0.10	5.49
5	41	12.78	-4.21	0.02	-7.64	-0.79	5.49	0.98	0.49	-1.82	3.78
6^\3^	40	14.90	-2.44	0.11	-5.47	0.58	4.65	0.44	0.76	-2.36	3.24
7	40	15.73	-0.99	0.54	-4.16	2.18	3.38	-1.72	0.24	-4.58	1.14
8	40	15.95	-0.58	0.72	-3.73	2.57	2.93	-2.18	0.12	-4.95	0.59
9	40	15.28	-1.00	0.56	-4.35	2.34	3.83	-1.52	0.31	-4.44	1.39
10	40	16.75	0.35	0.83	-2.86	3.55	3.28	-1.65	0.26	-4.50	1.19
11	40	14.48	-1.90	0.27	-5.29	1.48	4.65	-0.77	0.61	-3.75	2.20
12	40	15.18	-1.10	0.49	-4.22	2.02	5.40	-0.25	0.86	-3.16	2.65

1/ IG = index group, and CG = matching comparison group. The estimated mean difference is the bias-adjusted sample average treatment effect on the treated using nearest the neighbor matching estimator nnmatch in Stata 10, with 4 comparison workers matched to each index worker based on age, months of work experience, and gang.

2/ p-value for a z-test that mean difference = 0. 95% CI LL and UL are the lower and upper levels of the 95% confidence interval for the estimated average treatment effect on the treated.

3/ One index worker retired at the end of month 5.

**Table 3:** Index group means and matching estimator results for days plucking tea and non-plucking days for women

**Duration on ART**	**N**	**Days Plucking (IG Mean)**^\1^	**Estimated Mean Difference** **(IG – CG)**^\1^	**P > |z|**^\2^	**95% CI LL**^\2^	**95% CI UL**^\2^	**Days Non-Plucking Tasks (IG Mean)**	**Estimated Mean Difference** **(IG – CG)**^\1^	**P > |z|**^\2^	**95% CI LL**^\2^	**95% CI UL**^/2^
-24	56	15.46	-1.75	0.24	-4.68	1.19	5.09	1.48	0.20	-0.79	3.75
-23	56	15.61	-1.31	0.34	-3.99	1.37	4.50	0.17	0.87	-1.81	2.15
-22	56	15.59	-1.35	0.36	-4.27	1.57	4.43	0.94	0.43	-1.39	3.27
-21	56	16.68	-1.17	0.44	-4.15	1.82	2.54	-0.77	0.46	-2.77	1.24
-20	56	16.93	-0.49	0.72	-3.18	2.21	3.59	-0.68	0.51	-2.69	1.32
-19	56	14.07	-2.77	0.05	-5.56	0.03	4.27	0.03	0.98	-2.22	2.27
-18	56	15.52	-1.84	0.20	-4.67	0.98	6.02	2.01	0.10	-0.35	4.37
-17	56	17.25	-0.06	0.97	-2.66	2.55	3.68	0.27	0.78	-1.63	2.18
-16	56	17.48	0.18	0.88	-2.26	2.63	4.84	1.03	0.34	-1.09	3.15
-15	56	16.77	-0.11	0.94	-2.72	2.50	3.96	0.07	0.95	-2.05	2.19
-14	56	14.16	-3.49	0.01	-6.11	-0.86	5.32	1.75	0.12	-0.46	3.96
-13	56	16.88	-0.44	0.75	-3.11	2.23	5.30	1.48	0.21	-0.85	3.80
-12	56	16.13	-1.65	0.24	-4.41	1.11	4.21	1.21	0.28	-0.96	3.38
-11	56	16.91	-1.79	0.21	-4.60	1.02	3.63	0.85	0.43	-1.28	2.98
-10	56	16.48	-2.26	0.11	-5.00	0.48	3.95	1.26	0.26	-0.92	3.43
-9	56	14.23	-4.82	0.00	-7.69	-1.95	4.63	2.12	0.08	-0.25	4.50
-8	56	14.21	-5.39	0.00	-8.16	-2.62	5.54	2.66	0.02	0.41	4.91
-7	56	14.07	-4.68	0.00	-7.72	-1.64	4.39	2.19	0.04	0.06	4.32
-6	56	14.23	-4.99	0.00	-7.72	-2.27	4.07	1.36	0.18	-0.60	3.32
-5	56	13.95	-5.21	0.00	-8.09	-2.33	5.18	2.55	0.02	0.45	4.65
-4	56	13.98	-4.77	0.00	-7.40	-2.15	5.75	2.87	0.01	0.65	5.09
-3	56	13.89	-3.32	0.03	-6.24	-0.40	4.80	1.96	0.07	-0.19	4.11
-2	56	14.36	-3.37	0.02	-6.20	-0.54	4.39	2.34	0.02	0.31	4.38
-1	56	14.20	-4.61	0.00	-7.21	-2.01	3.88	1.69	0.08	-0.21	3.59
0	56	6.00	-13.55	0.00	-15.58	-11.53	4.05	2.04	0.02	0.34	3.75
1	56	7.45	-10.90	0.00	-13.50	-8.30	4.70	2.60	0.01	0.78	4.42
2	56	9.27	-8.14	0.00	-10.96	-5.32	6.70	3.98	0.00	1.60	6.35
3^/3^	55	9.73	-8.84	0.00	-11.36	-6.32	6.38	3.09	0.01	0.82	5.36
4	54	11.65	-6.63	0.00	-9.45	-3.82	6.63	3.56	0.00	1.31	5.80
5	54	10.44	-6.35	0.00	-9.24	-3.45	6.59	3.95	0.00	1.94	5.96
6	54	11.83	-5.93	0.00	-8.60	-3.25	6.43	3.66	0.00	1.52	5.81
7	54	13.19	-5.34	0.00	-7.96	-2.73	5.91	3.89	0.00	1.70	6.08
8	54	12.65	-6.14	0.00	-8.59	-3.69	5.93	4.17	0.00	2.21	6.13
9	54	12.43	-5.77	0.00	-8.42	-3.12	6.22	4.44	0.00	2.34	6.54
10	54	10.93	-6.62	0.00	-9.41	-3.84	6.91	4.19	0.00	2.00	6.38
11	54	13.33	-4.99	0.00	-7.65	-2.33	5.54	2.98	0.01	0.92	5.04
12	54	12.85	-5.69	0.00	-8.43	-2.95	5.61	2.68	0.02	0.49	4.86

1/ / IG = index group, and CG = matching comparison group. The estimated mean difference is the bias-adjusted sample average treatment effect on the treated using nearest the neighbor matching estimator nnmatch in Stata 10, with 4 comparison workers matched to each index worker based on age, months of work experience, and gang.

2/ p-value for a z-test that mean difference = 0, rounded to two digits. 95% CI LL and UL are the lower and upper levels of the 95% confidence interval for the estimated average treatment effect on the treated.

3/ One index worker retired at the end of month 2 and month 3.

**Table 4:** Index group means and matching estimator results for kilograms harvested when plucking for men and women

**Duration on ART**	**N** **(men)**	**Kilograms Harvested (IG Mean)**^\1^	**Estimated Mean Difference** **(IG – CG)**^\1^	**P > |z|**^\2^	**95% CI LL**^\2^	**95% CI UL**^\2^	**N** **(women)**	**Kilograms Harvested (IG Mean)**	**Estimated Mean Difference** **(IG – CG)**^\1^	**P > |z|**^\2^	**95% CI LL**^\2^	**95% CI UL**^/2^
-24	35	40.39	0.92	0.79	-5.98	7.83	47	34.24	-3.93	0.20	-9.96	2.10
-23	35	37.30	-1.26	0.70	-7.70	5.19	48	35.16	-5.72	0.08	-12.11	0.66
-22	34	37.36	-0.17	0.96	-6.51	6.17	51	36.35	-5.57	0.12	-12.52	1.38
-21	32	38.26	0.29	0.91	-4.92	5.50	54	35.07	-6.24	0.04	-12.24	-0.23
-20	32	41.21	-5.57	0.29	-15.96	4.82	54	31.99	-6.87	0.04	-13.36	-0.37
-19	35	45.71	-2.58	0.72	-16.74	11.58	50	33.79	-4.99	0.11	-11.10	1.12
-18	33	37.26	-1.69	0.59	-7.89	4.50	49	34.31	-5.37	0.04	-10.54	-0.20
-17	38	44.48	0.35	0.94	-8.02	8.72	53	32.76	-2.57	0.32	-7.64	2.51
-16	35	41.63	-1.94	0.59	-8.91	5.02	50	34.60	-3.53	0.19	-8.75	1.69
-15	36	38.71	-0.40	0.90	-6.51	5.71	50	29.38	-6.05	0.03	-11.63	-0.47
-14	36	41.54	-2.65	0.58	-11.97	6.67	49	28.19	-6.69	0.01	-11.53	-1.85
-13	36	35.22	-3.20	0.35	-9.95	3.55	49	31.79	-6.50	0.03	-12.38	-0.61
-12	36	43.47	0.67	0.88	-7.92	9.25	52	31.26	-5.36	0.10	-11.81	1.09
-11	36	44.35	4.05	0.30	-3.64	11.74	48	33.75	-1.86	0.53	-7.66	3.94
-10	36	41.84	-2.11	0.58	-9.47	5.26	49	31.94	-6.24	0.03	-11.77	-0.70
-9	34	40.91	-3.78	0.27	-10.48	2.92	45	33.26	-5.32	0.04	-10.32	-0.32
-8	36	35.68	-9.93	0.01	-16.92	-2.93	46	34.28	-6.05	0.03	-11.38	-0.72
-7	36	41.06	-8.36	0.06	-17.07	0.34	42	36.30	-4.10	0.14	-9.57	1.37
-6	36	43.75	-3.34	0.59	-15.33	8.65	42	34.17	-5.30	0.10	-11.64	1.04
-5	33	35.72	-5.35	0.16	-12.85	2.15	44	30.06	-6.72	0.07	-13.99	0.56
-4	33	42.27	2.35	0.60	-6.54	11.24	45	28.19	-8.28	0.02	-15.01	-1.55
-3	32	36.73	-3.78	0.28	-10.69	3.13	46	29.53	-10.96	0.00	-18.17	-3.74
-2	34	30.76	-11.21	0.00	-18.77	-3.65	47	33.40	-4.67	0.17	-11.29	1.95
-1	31	38.56	-6.47	0.11	-14.50	1.55	46	29.78	-8.80	0.01	-15.39	-2.21
0	29	37.29	-4.92	0.26	-13.49	3.65	39	27.59	-5.96	0.06	-12.23	0.31
1	24	35.71	-6.78	0.05	-13.43	-0.13	31	29.15	-5.08	0.12	-11.43	1.27
2	27	36.12	-10.40	0.01	-18.69	-2.11	33	26.74	-9.72	0.01	-16.42	-3.01
3	32	37.31	-1.14	0.73	-7.73	5.44	34	26.94	-7.10	0.00	-11.87	-2.34
4	34	36.96	-0.42	0.90	-6.87	6.03	39	29.19	-6.85	0.02	-12.39	-1.32
5	33	34.67	-6.59	0.03	-12.44	-0.74	40	33.13	-3.15	0.49	-11.99	5.70
6	36	37.20	-3.81	0.16	-9.07	1.46	43	31.30	-5.01	0.08	-10.62	0.60
7	37	36.47	-5.99	0.05	-11.98	-0.01	45	31.03	-4.32	0.07	-9.04	0.40
8	37	38.83	-1.91	0.65	-10.04	6.22	44	31.98	-6.10	0.01	-10.50	-1.69
9	37	35.52	-3.18	0.25	-8.56	2.20	39	35.62	0.87	0.75	-4.40	6.13
10	37	34.03	-3.00	0.22	-7.78	1.78	40	33.01	-3.81	0.21	-9.79	2.17
11	36	39.44	0.33	0.92	-6.37	7.04	38	32.19	-5.17	0.02	-9.54	-0.80
12	33	34.03	-2.04	0.42	-6.97	2.89	42	28.76	-5.49	0.02	-10.06	-0.92

1/ / IG = index group, and CG = matching comparison group. The estimated mean difference is the bias-adjusted sample average treatment effect on the treated using nearest the neighbor matching estimator nnmatch in Stata 10, with 4 comparison workers matched to each index worker based on age, months of work experience, and gang.

2/ p-value for a z-test that mean difference = 0, rounded to two digits. 95% CI LL and UL are the lower and upper levels of the 95%

### Results for men

Two years before initiating ART, the results reported in Table 2 and Table 4 show that the employment outcomes were not different for men in the index and comparison groups. Clearly circumstances and/or behaviours were different between the groups in the past, resulting in HIV infection, but these differences were not systematically related to employment outcomes.

During the entire pre-ART period (month -24 to -1), the male index group worked 1.84 fewer days plucking tea monthly than the matched comparison group (mean of 16.02 compared to 17.86; 10% less), although the estimated mean differences are statistically significant (p < 0.05) only for months -18, -8, -7, and -4. Men in the index group spent 1.24 more days on non-plucking tasks in the pre-ART period than the matched group (mean of 5.47 compared to 4.23; 23% more), but the estimated mean differences are significant only during months -17, -9, and -8. Men in the index group harvested 2.71 fewer kilograms when plucking (mean of 39.76 compared to 42.47; 6% less) in the pre-ART period than the matched comparison group, but the differences are significant only during months -8 and -2. The overall impact of HIV/AIDS on the work performance of the male index group in the 24 months prior to ART initiation thus appears to be modest.

Returning to Figure 1, after the substantial drop in days plucking during the month of ART initiation, the male index group continued to work substantially fewer days plucking tea than the matched comparison group during months 1–5 on ART (range 9.34–4.21 fewer days, p < 0.05 from Table 2). The number of days they spent plucking per month increased steadily, and from month 6 on ART no significant differences were observed (see Table 2). Other than in months 3 and 4 on ART, there were no significant differences in days working non-plucking tasks in the post-ART period. On days spent plucking, Table 4 and Figure 1 show that the male index group consistently harvested fewer kilograms per day than did those in the matched comparison group, although the differences were significant only for months 1, 2, 5, and 7. It thus appears that the work performance of male workers quickly improved after initiating ART and their employment outcomes were no longer distinguishable from the matched comparison group after 7 months on ART.

### Results for females

For women in the pre-ART period, the results reported in Tables 3 and 4 and illustrated in Figure 2 tell a substantially different story than for men. We estimated that the female index group worked about 25% fewer days per month than the matched comparison group beginning in month -9 and continuing through month -1 (monthly range: -3.32 to -5.21 fewer days; p-value < 0.05 for each month). To compensate to some degree for the lost income from working fewer days harvesting tea, the female index group worked about 87% more days on non-plucking assignments during months -8 through -1 than the comparison group (monthly range: 1.36 to 2.86 more days; p-value < 0.10 for each month). The index group also harvested 15% less tea when plucking than the comparison group during the pre-ART period (monthly range: 1.86 to 10.96 fewer kilograms; p-value < 0.05 for 14 out of 24 pre-ART months).

After initiating ART, women in the index group continued to work fewer days plucking tea and more days on non-plucking assignments than the comparison group (monthly ranges: 10.90 to 3.87 fewer days plucking, 2.60 to 4.44 more non-plucking days; p-value < 0.05 for all months, both outcomes). While the positive trend in days plucking observed during months 1 to 12 is encouraging, the female index group still worked 30% fewer days plucking tea than the female comparison group by the 12^th ^month on ART. Women in the index group also generally harvested fewer kilograms on days spent plucking than did the comparison group throughout the study period (p-value < 0.05 for 7 of 12 post-ART months).

### Relationship to Prior Research

We previously reported preliminary results for one outcome, days plucking tea, for 59 index subjects [26] based on a simple comparison of means between the index group and a comparison group matched only on gang. These 59 index subjects worked significantly fewer days plucking tea monthly than the comparison population beginning in the 9^th ^month pre-ART. After initiating ART, they quickly increased days plucking during their initial 12 months on therapy, although by month 12 they continued to work 2.67 fewer days (p-value = 0.04) than the comparison group.

The analysis and results reported in the current analysis provides a substantial improvement to the earlier analysis. First, due to the smaller sample size (33 women and 26 men in the index group) in [26], we did not stratify the analysis by gender. The results reported in this analysis show that stratifying by gender is needed both to understand dynamics in the intervention group as well as the general workforce. Second, beyond gender, matching a fixed number of comparison workers in the same gang based on age and experience creates a better comparison group for analysis. Since some gangs have larger numbers of workers than others, the comparison group in the earlier analysis was weighted more heavily to larger gangs. And third, while days plucking tea is one employment outcome of interest, the current paper includes two other key outcomes, kilograms harvested and days working non-plucking assignments, that provide a more complete picture of employment adjustments over time during the pre- and post-ART periods.

## Conclusion

Using payroll and employment records from a large tea plantation in Kericho District of Kenya, we examined the employment patterns of 97 HIV-infected workers (56 women and 41 men) receiving ART at their company clinic from two years pre-ART through 12 months post-ART. We found that male index workers were able to maintain a similar pattern of work as the male comparison group until the month they initiated therapy and then returned to a similar work pattern by their 7^th ^month on ART. For women, we found evidence of substantial differences in employment outcomes, mainly through being less productive while plucking, working fewer days plucking tea, and shifting to non-plucking work assignments. Although women in the index group increased the number of days they spent harvesting tea once they initiated ART, they continued to spend fewer days plucking tea in the 12^th ^month on ART. To compensate, they worked substantially more days on other non-plucking tasks than the general female workforce.

There are probably multiple reasons for the differences in the employment outcomes observed between HIV-infected women and men in the pre-ART and post-ART periods. There is little evidence in the literature of gender-related biological differences in either disease progression or the effectiveness of ART, though the available research comes from industrialized country settings [27]. In some studies in southern Africa, women face a greater risk of ARV-related toxicities, particularly hyperlactatemia and lactic acidosis associated with d4T [28], though this finding was not replicated in research in Kenya [29].

Some of the differences in gender-specific employment outcomes we observed are likely to be associated with socioeconomic factors. Women may experience more HIV-related morbidity and/or fatigue than men in the pre- and post-ART period as a result of the greater household demands on their time. Women typically allocate much of their non-wage earning time to household production activities, such as cooking, cleaning, child care, and care for sick family members. The latter may be particularly important for women in our index population, who may well have HIV-positive children or spouses. Staff at the ART clinic may be more likely to request or recommend that their female patients be assigned to non-plucking days more than their male patients.

In the absence of antiretroviral therapy, HIV-infected individuals eventually develop AIDS and die. While the progression of the disease from HIV-infection to an AIDS diagnosis may be 10 years or more, studies from resource-limited settings suggest a median survival time of 11 months following an AIDS diagnosis [7]. Thus, if the tea pluckers included in this study had not initiated therapy, some 50% would likely have died by the end of the study period. Instead of dying, however, the results presented in this paper show that HIV-infected workers who accessed ART in this resource limited setting returned to physically demanding, labor intensive activities after starting treatment.

The number and representativeness of the workers in the index group are a potential limitation of this study for a broader generalization to the treated population in this study site. Regarding numbers in the index groups, it is possible that the male index group (n = 41) is too small to detect significant mean differences given the relatively small size of the estimated differences. Future analysis with a large number of workers in the index group and for a longer follow up period is needed to explore this issue further. Regarding representativeness, enrolment in the study began in March of 2006 as patients came to the central ART clinic for regularly scheduled clinic visits. We cannot determine whether the index group constitutes a random sample of the ART-eligible population of tea pluckers, estimated at about 150 total tea pluckers at the time of enrolment. A few potential participants (<5) discussed enrolment with the study nurse but declined to participate. After consenting, no one in the index group died during the time period included in this analysis.

Although this study is being conducted in a plantation setting, the benefits of ART observed in this setting are relevant, at least to some extent, to the general rural population of Kenya. Plucking tea is physically demanding outdoor work. Pluckers walk substantial distances to fields early in the morning, stand for hours with heavy baskets on their backs, push through rows of bushes, carry heavy packs to weighing stations, and repeat numerous times a day the process of reaching, plucking, raising arms, and placing tea leaf in their basket. Many of the characteristics of tea plucking are thus similar to those of other types of labor commonly found in rural Kenya, such as family labor on one's own farm. On a family farm, there are often several types of tasks to be performed on the same day, so that individuals can shift among tasks depending on their health.

Two clear differences exist, however, between the tea estate workers and other rural workers. First, relative to informal sector workers, tea pluckers are not especially poor or malnourished and generally have good access to high quality health care on the plantation. This is clearly not the case for some portion of the rural population. Second, barriers to accessing and adhering to ART within this plantation setting are probably as low as possible. Cash transportation costs to the ART clinic are zero (the company provides transportation) and travel times are modest.

## Competing interests

The authors declare that they have no competing interests.

## Authors' contributions

BAL contributed to study conception and design, acquisition of data, data analysis and interpretation, and drafting and critically revising the manuscript. MPF contributed to study conception and design, acquisition of data, data analysis and interpretation, and drafting and critically revising the manuscript. SR contributed to study conception and design, interpretation of results, and drafting and critically revising the manuscript. MB contributed to study design, acquisition of data, interpretation of results, and critically revising the manuscript. CS contributed to study design, acquisition of data, interpretation of results, and critically revising the manuscript. DS contributed to study design, acquisition of data, interpretation of results, and critically revising the manuscript. FS contributed to study design, acquisition of data, interpretation of results, and critically revising the manuscript. KM contributed to study design, interpretation of results, and critically revising the manuscript. MW contributed to study conception and design, acquisition of data, interpretation of results, and critically revising the manuscript. JLS contributed to study conception and design, acquisition of data, interpretation of results, and critically revising the manuscript. All authors read and approved the final manuscript.

## Pre-publication history

The pre-publication history for this paper can be accessed here:


